# Targeting trafficking as a therapeutic avenue for misfolded GPCRs leading to endocrine diseases

**DOI:** 10.3389/fendo.2022.934685

**Published:** 2022-08-25

**Authors:** Alfredo Ulloa-Aguirre, Teresa Zariñán, Rubén Gutiérrez-Sagal, Ya-Xiong Tao

**Affiliations:** ^1^ Red de Apoyo a la Investigación (RAI), National University of Mexico and Instituto Nacional de Ciencias Médicas y Nutrición SZ, Mexico City, Mexico; ^2^ Department of Anatomy, Physiology & Pharmacology, Auburn University College of Veterinary Medicine, Auburn, AL, United States

**Keywords:** G protein-coupled receptors, GPCRs, intracellular trafficking, quality control system, molecular chaperones, pharmacological chaperones, pharmacoperones

## Abstract

G protein-coupled receptors (GPCRs) are plasma membrane proteins associated with an array of functions. Mutations in these receptors lead to a number of genetic diseases, including diseases involving the endocrine system. A particular subset of loss-of-function mutant GPCRs are misfolded receptors unable to traffic to their site of function (*i.e.* the cell surface plasma membrane). Endocrine disorders in humans caused by GPCR misfolding include, among others, hypo- and hyper-gonadotropic hypogonadism, morbid obesity, familial hypocalciuric hypercalcemia and neonatal severe hyperparathyroidism, X-linked nephrogenic diabetes insipidus, congenital hypothyroidism, and familial glucocorticoid resistance. Several *in vitro* and *in vivo* experimental approaches have been employed to restore function of some misfolded GPCRs linked to endocrine disfunction. The most promising approach is by employing pharmacological chaperones or pharmacoperones, which assist abnormally and incompletely folded proteins to refold correctly and adopt a more stable configuration to pass the scrutiny of the cell’s quality control system, thereby correcting misrouting. This review covers the most important aspects that regulate folding and traffic of newly synthesized proteins, as well as the experimental approaches targeted to overcome protein misfolding, with special focus on GPCRs involved in endocrine diseases.

## Introduction

G protein-coupled receptors (GPCR) and their associated signaling modules represent one of the major systems employed by the cells to communicate with each other and transmit information across long and short distances within the body. Sensory receptors for light, taste, and smell also belong to the GPCRs superfamily (1–3). GPCRs constitute the largest family of membrane receptors in many animal species, including humans. In fact, 4-5% of the human genome codes for these important plasma membrane proteins (1, 3). The importance of GPCRs and GPCR ligands in human disease is supported by the fact that both still continue to be the focus for new drug discovery (currently in the form of small molecules and peptides), despite being known as druggable targets for a long time. Indeed, it is estimated that approximately 30-40% of approved drugs target this family of membrane receptors (4–6).

Although GPCRs widely vary in molecular size (7–9), they share a common structure conformed by a single, serpentine-like, polypeptide chain that crosses the plasma membrane (PM) seven times forming transmembrane domains (TMD), which are hydrophobic, α-helix structures connected by extracellular (EL) and intracellular (IL) loops, with an ectodomain (ECD) and an intracellular carboxyl-terminal (COOH) domain also called C-tail (7, 9). These cell surface plasma membrane-embedded receptors exist in complex with one or several heterotrimeric G proteins and also associate with membrane-linked intracellular proteins that activate upon ligand binding and that regulate activation of a number of G protein-dependent and -independent signaling cascades (7, 9, 10). Upon activation, GPCRs are desensitized and internalized through the formation of endosomes, where a second wave of signaling may occur or terminate, and the destiny of the internalized receptor is defined (11–13). Therefore, the net amount of a particular GPCR expressed at the PM that is exposed to extracellular messengers, including the cognate ligand, will depend on its dynamics of intracellular trafficking from the endoplasmic reticulum (ER), where it is synthesized, to the its final destination at the PM, the fate of the receptor following agonist-stimulated internalization (degradation *vs.* recycling), and the normal membrane turnover (13). Since GPCRs participate in an array of functions, it is not surprising that they are activated by a number of structurally diverse ligands that include photons, ions, odorants, lipids, peptide and non-peptide hormones, and several neurotransmitters that vary in size (7–9). Approximately 50% of GPCRs bind endogenous ligands, whereas the remaining receptors sense the chemical environment (1, 14). Therefore, in complex organisms, GPCRs are intermediate stations that communicate the internal and external environments of cells.

Mutations in GPCR genes may alter receptor synthesis or function in a number of genetically- determined disorders (15). Loss-of-function GPCR mutations may affect the amino acid sequence of the protein and lead to alterations in domains critical for recognition and binding of agonist, receptor activation and/or coupling to G proteins, or internalization. They also may cause errors in the three dimensional configuration of the receptor protein (*i.e.* misfolding) due to non-native interactions that may impact in a significant manner not only the overall architecture of the protein but also its physicochemical characteristics, leading to its intracellular retention (13, 16–20). Protein misfolding can also occur as the result from protein overexpression, thermal or oxidative stress, and activation of pathways involved in the regulation of protein folding, maturation, and quality control (16). Although some non-specific, non-native interactions with other intracellular peptide or proteins may occur during the normal dynamics of folding (to mask aggregating-prone regions), abnormally folded proteins may accumulate as toxic aggregates and form extracellularly deposited amyloids (21–23).

Recent studies on how the folding process occurs have provided important information for the design of pharmacological interventions that may correct misfolding of defective proteins that cause disease. The finding that some alterations in the amino acid sequence of a protein leading to misfolding do not involve domains essential for its function has further open the way for discovering new therapeutic avenues, including pharmacologic strategies to correct abnormal folding and routing to the PM of mutant misfolded ion channels (*e.*g. the cystic fibrosis transmembrane conductance regulator protein -CFTR-) and GPCRs (see below) (20, 24–33).

The present review focuses on GPCR trafficking, misfolding, and misrouting as a cause of endocrine disorders and how different genetic, physical and pharmacological strategies may rescue, partially or completely, function of GPCRs with folding defects. To better understand these aspects, let’s briefly review how the cell quality control system works to preserve a stable and functional proteostasis within the cell, with particular focus on GPCRs.

## The quality control system (QCS)of the cell and the role of molecular chaperones

**Figure 1:**
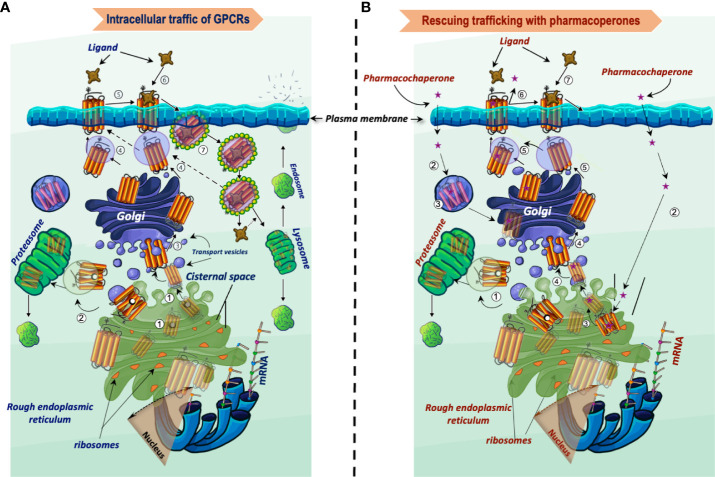
Intracellular trafficking of GPCRs belonging to the rhodopsin-like family of receptors. **(A)** Newly synthesized proteins fold in the endoplasmic reticulum (step 1), where misfolded proteins interact with molecular chaperones and co-chaperones (oval black and white structures, respectively), which attempt to correct folding and stabilize the protein in a conformation adequate for endoplasmic reticulum export. When correction of misfolding fails, the abnormal protein is dislocated into the cytoplasm for proteasomal degradation (step 2). Folded GPCRs are translocated to the Golgi apparatus to complete their maturation process including glycosylation (arbor-like structure within the magnifier). Mature receptors then traffic to the plasma membrane where they bind their cognate ligands (steps 4 to 6). Following activation of the receptor by agonists, phosphorylation and recruitment of β-arrestins occur, which provoke endocytosis and internalization of the receptor–ligand complex (step 7). The internalized complex is embedded in clathrin-coated vesicles, which may be either targeted to lysosomes for degradation or dissociate with subsequent sorting of the receptor to the recycling pathway (step 4). **(B)** Rescue of misfolded receptors by pharmacoperones. Misfolded/misrouted receptors that could not be stabilized by molecular chaperones are submitted to degradation (step 1). The pharmacoperone drug crosses the cell surface plasma membrane, penetrates into the cell (step 2) and specifically binds the misfolded GPCR (step 3). Receptors stabilized by the pharmacoperone are then exported to the Golgi apparatus for further processing (step 4), and finally to the plasma membrane (step 5); here, the pharmacoperone (in the case of antagonists or agonists of the receptor) must dissociate from the rescued receptor for allowing the agonist to recognize and bind the rescued receptor promoting its activation (steps 6 and 7). Interaction of pharmacoperones with intracellularly trapped receptors also may occur in compartments other than the ER before degradation (step 3, left) (46).

The QCS of the cell is a complex molecular machinery that continuously surveys and monitors newly synthesized proteins at the ER, Golgi and the PM (34–36). The molecular chaperones and companion factors (*e.g.* co-chaperones, modification enzymes, and targeting factors) are key elements of this QCS that play an essential role in maintaining the integrity of the proteostasis network (35, 37–39). Molecular chaperones are an essential driving force in the highly dynamic and complex folding process of the nascent proteins occurring within a crowded and viscous environment of proteins accumulated in the ER. Molecular chaperones are resident proteins involved in the co- and posttranslational regulation of several processes occurring during protein synthesis, including folding, assembling, and degradation of defective proteins; they also regulate ER exit and prevent toxic accumulation and aggregation of misfolded intermediates (37, 40–42). When proteins fail to achieve proper folding and an appropriate minimal-energy configuration, the QCS will promote the export of the conformationally defective protein to the degradation pathway, mainly the ubiquitin-proteasome system (43–45). Contrariwise, achievement of a conformation compatible with ER export will lead to translocation of the protein to the Golgi apparatus to complete processing and maturation (**Figure 1A**). Surveillance of the QCS relies more on its capacity to detect misfolding of the nascent proteins based on non-native structural determinants including exposure of hydrophobic shapes, unpaired cysteines or immature glycans, and specific amino acid sequences or motifs involved in the regulation of protein trafficking within the cell (37).

Major molecular chaperones represent a key machinery for quality control [e.g. the heat shock proteins of 70 kDa and 90 kDa (HSP70 and HSP90, respectively)]; these chaperones assist proteins in folding and remodeling (47–49). HSP70 ATP-dependent molecular chaperones are highly conserved and the best studied heat shock proteins (49). Nascent polypeptides are recognized by HSP70 chaperones, which promote their folding, stabilization, translocation, and degradation when necessary (48–52). One of the most important folding factors is BiP/Grp78 which is a member of the HSP70 family of proteins of the endoplasmic reticulum (47, 53–55) and a key folding factor of the ER. Several GPCRs interact with BiP; these include the luteinizing hormone/chorionic gonadotropin receptor (LHCGR), the angiotensin II AT_1_ receptor, rhodopsin, and the thyroid-stimulating hormone (or thyrotropin) receptor (TSHR) (56–59). This chaperone is assisted by the DnaJ family members of cofactors (HSP40) to recognize hydrophobic regions during the protein folding process, aiding to keep proteins in a conformation permissive for continuing folding and oligomerization (55). Among the roles of nucleotide-bound BiP is on the protective unfolded protein response (UPR), which is an important stress reaction that decreases unfolded protein load to maintain proteostasis, cell viability, and function during cellular stress (60, 61).

Calnexin/calreticulin and protein disulfide isomerases also belong to the major chaperone family. Calnexin/calreticulin works on *N*-linked carbohydrates and unfolded regions in the protein, assisting in folding through the so-called calnexin/calreticulin cycle (62–66). Meanwhile, the protein disulfide isomerases (PDI), such as the oxidoreductase PDI, decreases the thermodynamic stability of the protein by introducing disulfide bridges, thereby promoting reorganization of folding intermediates (67–70). Several GPCRs, associate with calnexin and calreticulin; in this vein, mutations that impair protein glycosylation may result in misfolding, which when detected by the ER-QCS, limits the anterograde traffic of the misfolded conformer to the PM (71). PDI works as a co-chaperone with calnexin and calreticulin during their association with glycoproteins (72). Their study have allowed to recognize differences in folding between species of particular mutant proteins such the inactivating Ala593Pro and Ser616Tyr mutants of the LHCGR, which lead to male pseudohermaphroditism; patients with this disorder exhibit severely impaired response to LH and hCG due to misfolding and intracellular trapping of the mutant receptors [reviewed in (73)]. Similar to the wild-type (WT) LHCGR, the Ala593Pro and Ser616Tyr LHCGR mutants were detected associated with calnexin, whereas the WT receptor interacted with PDI. Although both of this receptor mutants appeared to interact with BiP/Grp78, only Ala593Pro was found to associate with Grp94 (74). Their distinct association with molecular chaperones (57) suggests that these mutant receptors differ in conformation.

Other chaperones (called *non-classic private chaperones*) and interacting proteins also associate with GPCRs during their upward trafficking. As these interacting proteins regulate protein folding and trafficking in different ways, they may be considered as molecular chaperones with a specific function on particular GPCRs [reviewed in (75)]. Among these non-classic chaperones are: *a.* ANKRD13 (ankyrin repeat domain-containing protein 13C); GPCRs that interact with this chaperone include the prostaglandin D_2_ receptor, the thromboxane A_2_ receptor, the chemoattractant receptors, and the β_2_-adrenergic receptor (β_2_AR) (76); *b.* Ribophorin I, a chaperone that interacts with the *μ*-opioid receptor (77); *c.* ATBP50 (angiotensin II receptor-binding protein of 50 kDa), which associates with the C-tail of the angiotensin II AT2 receptor (78); *d.* Golgin-160 (which localizes in the Golgi) and gC1q-R (receptor for globular heads of C1q), which promote upward trafficking of the β_1_AR and α_1B_AR, respectively (79); *e.* Receptor activity modifying proteins (RAMPs) interact with a number of GPCRs from different classes, including the calcitonin-receptor-like receptor, the corticotropin-releasing factor 1 receptor, and the calcium-sensing receptor (CaSR), promoting their translocation to the PM as well as desensitization and recycling of particular GPCRs (80); *f.* RanBP2, which binds red/green opsin molecules (81); g. The molecular chaperone ODR4, facilitates folding, exit from the endoplasmic reticulum, and PM targeting of a subset of olfactory GPCRs, such as such as ODR10 in *C. elegans* and rat U131 in undifferentiated olfactory-derived odorant receptor activatable (odora) cells and Chinese hamster ovary cells (82–84). Its homolog in humans, C1orf27, interacts with the α_2_A-AR to regulate its anterograde traffic (85); h. Melanocortin-2 receptor (MC2R) accessory protein (MRAP), which enhances MC2R traffic (86, 87); i. RTP1 and RTP2, which interact with odorant receptors, and enhance responses to odorants (88); and *j.* DRiP78, the ER-membrane protein, dopamine receptor interacting protein of 78 kDa, which associates to the F(*x*)_3_F(*x*)_3_F hydrophobic motif at the proximal COOH-terminus of α-helix8 of several GPCRs (89, 90)

Molecular chaperones and the chaperone network at the ER and the Golgi as well as the peripheral quality control checkpoints [see for review (91)], are attractive therapeutic targets for exogenously regulating protein trafficking and secretion (92–94).

## Structural determinants and GPCR-dependent factors that regulate anterograde trafficking from the ER to the plasma membrane

Several sequences present in GPCRs have been identified associated with components of coat complex protein II transport machinery and Ras-related small GTPases necessary to allow the protein to exit the ER and enter the ER-Golgi intermediate compartment (90, 95–98). These motifs are particularly localized in the C-tails and less frequently in the loops and ectodomain of GPCRs, and include the above mentioned F(*x*)_3_F(*x*)_3_F motif identified in helix-8 of the angiotensin II AT_1_ receptor, the dopamine D_1_ receptor, and the M_2_-muscarinic receptor (99), and the E(*x*)_3_LL and FN(*x*)_2_LL(*x*)_3_L leucine motifs in the human vasopressin 2 (V_2_R) and vasopressin 3 (V_3_R) receptors, respectively (100–103). Alterations in these motifs lead to trapping of the protein and hence markedly impact receptor export to the PM. Some GPCRs employ these motifs for anterograde trafficking from the trans-Golgi network to the cell surface PM through association with the small GTPase Rab8 (97). The export sequence F(*x)*_6_LL motif identified in the proximal NH_2_-terminal end of the C-tail of several GPCRs (104, 105), including the gonadotropin receptors, the angiotensin AT_1_ receptor, and the α_2B_-, α_1B_-, and β_2_-ARs, is an important motif that controls the upward trafficking of GPCRs. The location of this latter motif is important given the known interaction of DRiP78 with the overlapping conserved (hydrophobic) sequence F(*x*)_3_F(*x*)_3_F present in several GPCRs; alteration in this motif leads to intracellular retention of the receptor (89). Modifications in retention motifs that limit GPCR ER exit for further processing may *improve* trafficking of the cargo protein. Some of these motifs are: *a.* the RSRR sequence in the GABA-B1 receptor (106); *b.* the 5R (penta-arginine) sequence in the α_2C_AR (107); and *c.* the conserved ALAAALAAAAA hydrophobic sequence in the extracellular NH_2_-terminal region of the α_2_-AR (108). These retention signals probably act by limiting intracellular traffic of receptors that do not hide retention motifs as a consequence of misfolding or that fail to heterodimerize (109). Therefore, export and retention signals are mechanisms that regulate that only adequately folded and assembled receptor complexes are exported to the PM.

Two highly conserved motifs present in GPCRs belonging to the rhodopsin/β-adrenergic-like receptors are important, the E/DRY motif at the boundary of the α-helix3 and the IL2 and the N/DP*xx*Y motif at α-helix7, near its cytoplasmic face of the PM. Alterations in these sequences may lead to misrouting, depending on the particular GPCR [*e.g.* alteration on both motifs in the gonadotropin-releasing hormone receptor (GnRHR) and the V_2_R, and on the N/DP*xx*Y motif in the endothelin-B receptor, melanocortin-4 rreceptor (MC4R), and the CC chemokine receptor 5 (CCR5) (110, 111)]. Another important structural feature of GPCRs belonging to rhodopsin/β-adrenergic-like family of GPCRs is a disulfide bond between the EL1 and EL2, which seems necessary for stabilization of the TMD structure. Alterations in or around this disulfide bridge severely alter the 3-dimensional structure of the receptor, and lead to intracellular retention and degradation of the receptor protein. In fact, mutations at this particular location make the human GnRHR difficult to stabilize with pharmacological chaperones (see below) (111).

Few export motifs in the loops and ectodomain of GPCRs have been identified. A distinct YS motif in the NH_2_-terminus of both the α_2A_- and α_2B_-AR has been identified as an export motif from the Golgi apparatus (112). A single and highly conserved leucine residue at the center of the IL1 seems to play an important role in ER export of several adrenergic receptors and the angiotensin II AT_1_ receptor (113), and lastly, a triple arginine (3R) motif in the IL3 mediates interaction of the α_2B_AR with protein transport Sec24C/D isoforms (95).

Glycosylation and palmitoylation are frequent posttranslational modifications that in some GPCRs regulate anterograde GPCR traffic. *N*-linked glycosylation at the consensus sequence N-*x-*S/T is a frequent modification that helps folding by increasing the solubility of the protein and enhancing its conformational stability (63, 114). When *N*-linked glycosylation or early glycan processing is altered, misfolded glycoproteins are identified by the QCS blocking further anterograde trafficking to the PM (71). Mutations at or close to glycosylation sites in GPCRs (in which glycosylation in their ectodomain is required for anterograde export), may lead to reduced PM expression (115, 116). Nevertheless, it is important to note that that alterations in the structure of the receptor ectodomain bearing particular glycosylation sites may also hamper folding and thereby lead to limited PM expression. Meanwhile, the reversible addition of fatty acids to the cysteine residues in the C-tail of several GPCRs is another frequent posttranslational modification (117). This modification anchors the receptor to the PM forming a fourth intracellular loop (117), and also regulates several functions of the receptor, including upward trafficking, coupling to G proteins, β-arrestin recruitment, and the fate of the internalized receptor (118–121).

It has been shown that a number of GPCRs associate in multi-unit complexes during their biosynthesis or processing in the Golgi (122). Oligomerization as an effective quality control of protein folding before export to the PM has been demonstrated in a number of GPCRs (122–132). A typical example is the association between the GABA-B receptor-1 and GABA-B receptor-2, which is apparently an obligatory requisite for cell surface PM expression of a functional receptor; in this particular GPCR, association between the C-tails of these GABA-B receptors hides a retention motif (*e.g*. the R*x*R ER retention signal in the GABA-B1 receptor C-tail), thereby promoting export of the heterodimer to the PM (133). In this vein, mutations in GPCRs may provoke dominant negative effects *via* protein-protein association, thereby interfering with WT and/or mutant receptor upward trafficking and PM expression (131). This effect of mutant receptors on upward trafficking occurs in several GPCRs (134–139) and might have a physiopathogenic role in the phenotypic expression of disease in individuals carrying simple heterozygous mutations.

In ensemble, upward trafficking of GPCRs through the secretory pathway relies on several factors, including: *a.* The molecular chaperones of the QCS of the cell; *b.* Retention and export motifs present within the nascent GPCR protein; *c.* The well-organized association and interaction between GPCRs; and *d.* Particular posttranslational modifications involved in export of the GPCR from the ER to the Golgi and from the latter to the PM, as well as on the post-endocytic fate of the receptor after agonist-stimulated internalization.

## Misfolding of GPCRs as a cause of endocrine disorders

A wide variety of genetic diseases in humans occurs as a result of mutations in GPCR genes, some of which lead to defects in receptor folding and intracellular trapping of the receptor protein. Examples of endocrine diseases caused by GPCR misfolding and misrouting are shown in **Table 1**. These include a significant number of Class II loss-of-function mutants [which lead to intracellular retention of receptors due to misfolding of the protein (20, 162)] causing familial hypocalciuric hypercalcemia and neonatal hyperparathyrodism provoked by mutations in the CaSR (calcium-sensing receptor), hypogonadotropic hypogonadism [mutations in the GnRHR and the prokineticin receptor 2 (PROKR2) genes], X-linked nephrogenic diabetes insipidus (mutations in the V_2_R gene), congenital hypothyroidism (mutations in the TSHR gene), morbid obesity caused by mutations in the MC4R gene, adrenocorticotropic hormone (ACTH) insensitivity with adrenal insufficiency (mutations in the MC2R gene), and reproductive disorders [LHCGR and follicle-stimulating hormone receptor (FSHR) gene mutations], among others (73, 75, 140, 149, 157, 163, 166–168) (**Table 1**). Diseases caused by mutations that lead to GPCR misrouting are of particular interest because they may be specifically treated with drugs that correct trafficking and rescue function of the mutant receptors, whenever critical domains involved in essential functions (*i.e*. binding to agonist, activation of the receptor and/or coupling to effectors) are not compromised by the mutation. Some examples of drugs whose development is based on targeting trafficking of misfolded, mutant GPCRs in endocrine diseases include small molecules designed for rescuing function of the V_2_R, MC4R, GnRHR, and more recently gonadotropin receptors (169–171). The discovery of this pharmacological approach suggests that by screening drugs for treating variant GPCRs may efficiently detect novel therapeutic approaches (172). Probably one of the most notable examples of pharmacological rescue of misfolded membrane proteins is the case of the ΔF508 defective cystic fibrosis transmembrane conductance regulator -CFTR- for which several pharmacoperones have succeeded in restoring membrane expression and function of the mutant Cl^-^ channel in individuals with cystic fibrosis (25, 28–33).

**Table 1:** Endocrine diseases caused by misfolded, trafficking defective GPCRs.

Disease	GPCR involved	Reference
**X-linked nephrogenic diabetes insipidus**	Vasopressin 2 receptor (V_2_R)	(140–148)
**Hypogonadotropic hypogonadism**	Gonadotropin-releasing hormone receptor (GnRHR), prokineticin receptor 2 (PROKR2)	(73, 140, 149–152)
**Familial hypocalciuric hypercalcemia**	Calcium-sensing receptor (CaSR)	(153–156)
**Hypergonadotropic hypogonadism**	Luteinizing hormone receptor and follicle-stimulating hormone receptor (LHCGR and FSHR, respectively)	(73)
**Congenital hypothyroidism**	Thyrotropin receptor (TSHR)	(157–159)
**Adrenal insufficiency**	Melanocortin-2 receptor (MC2R)	(160, 161)
**Obesity**	Melanocortin-3 and -4 receptors (MC3R and MC4R)	(75, 162–165)

## Experimental strategies to rescue misfolded GPCRs

**Figure 2:**
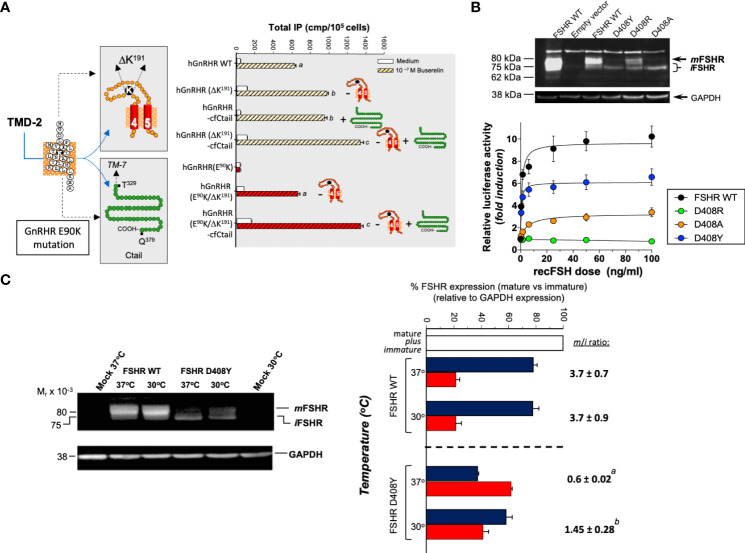
The effects of genetic **(A, B)** and physical **(C)** maneuvers on the expression and function of misfolded mutant human (h) GnRHR and hFSHR *in vitro*. **(A)** *Left image:* The E90K mutation at the TMD2 of the hGnRHR leads to hypogonadotropic hypogonadism and provokes misfolding and intracellular trapping of the receptor protein (169). Two genetic modifications *rescued* membrane expression and function of the receptor: (*a*) Deletion of a lysine residue at position 191 in the EL2, which connects TMDs 4 and 5 (K191 is absent in the rat GnRH receptor, which exhibits a high membrane expression of the corresponding receptor); and (*b*). Addition of the C-tail of the catfish GnRHR (the C-tail is absent in the hGnRHR) (*left image*). In the *right graphic* of A, it can be observed how deletion of K191 (-) in both the WT and mutant E90K GnRHR and/or addition (+) of the C-tail from the catfish GnRHR increased total inositol phosphates production upon stimulation with the GnRH analog Buserelin. Different letters above bars indicate statistically significant differences among inositol phosphate values. Data taken from ref (169).**(B)** This figure shows the effect of different amino acid replacements on the hFSHR at position D408 (D408Y, D408R, and D408A) in the TMD2, on the plasma membrane expression of the receptor (*top*) and activation of the cAMP-sensitive pSOMLuc reporter plasmid (*bottom*). *Top*: Western blot of hFSHR WT and mutants (D408Y, D408R, and D408A, respectively) hFSHRs. The blot shows the migration of hFSHRs from protein extracts of HEK293 cells transiently transfected with the WT (lane 3) or mutant hFSHR (lanes 4-6) cDNAs inserted in the pSG5 vector. The first lane from left to right shows the migration of the WT hFSHR from HEK293 cells stably expressing the receptor. The immunoblot shows that the naturally occurring mutant D408Y is mainly detected as an intracellular, immature (i) form of the receptor [≤ 75 kDa], whereas in the case of the D408R, laboratory-manufactured mutant, the replacement leads to an FSHR molecule that is present as both mature [plasma membrane expressed, ~80 kDa; (m)] and immature forms, albeit the expression of the mature form is lower compared with that of the WT hFSHR (lane 3). In the case of the D408A mutant, the expression of the mature form is marginal and that of the immature form predominates. *Bottom:* Recombinant FSH-stimulated intracellular signaling of the WT and the D408Y, D408R, and D408A hFSHR mutants, as assessed by a reporter gene assay, in HEK293 cells transiently cotransfected with the WT or mutant hFSHRs and the cAMP-sensitive pSOMLuc reporter plasmid. The results showed that the WT hFSHR induced a robust dose-dependent response in luciferase activity, whereas the D408Y and D408A mutants showed markedly reduced responses to FSH stimulation, and the D408R mutant showed virtually absent response to agonist. Thus, replacement of aspartate with arginine at position 408 rescued membrane expression but not function of the receptor, whereas replacement with alanine was ineffective in restoring PM expression and marginally effective in rescuing function of the mutant hFSHR (from ref (174). **(C)** The effect of reducing the incubation temperature of HEK293 cells transiently expressing the WT and mutant D408Y hFSHR, on the plasma membrane expression of the FSHRs and the trafficking defective D408Y mutant. Incubation at a reduced temperature (30°C) enhanced PM expression of the mutant D408Y receptor (*left immunoblot*), with the corresponding increase in the mature (m) to immature ratio (i) (*m/i* ratio) (*right graph*). From ref (176).

Among the experimental strategies that may promote rescue of misfolded GPCRs *in vitro* and/or *in vivo* are genetic, physical, chemical, and pharmacological approaches. Genetic strategies for increasing PM expression of misfolded proteins are intended to introduce or delete particular sequences into the mutated protein (173–175). By using this approach, the defective receptor may be overexpressed or conformationally stabilized without global changes in the ER secretory activity. This may be achieved, for example, by adding residues or motifs for glycosylation at the NH_2_-terminal domain of the receptor protein or COOH-terminal sequences to expression-deficient GPCRs, thereby markedly enhancing ER export to the PM. In some cases, the genetic modification increase PM expression of the misfolded receptor (**Figure 2A**), but also may affect receptor activation because of the conformational modification in the domain involved in this particular function (174) (**Figure 2B**). In others, such as the P320L human GnRHR mutant, the abnormal protein is unrescuable by genetic approaches because proline´s peptide backbone is constrained in a ring structure; the presence of proline is associated with an imposed turn in the sequence of the α-helix in TMD7, whose structure is severely disturbed when replaced. One example of efficient genetic rescue is the mammalian GnRHR; this particular receptor lacks the intracellular C-tail extension and by adding this domain from the catfish GnRHR or by deleting de arginine residue at position K191 [whose presence limits PM expression of the primate receptor (177)], significantly increased cell surface PM expression (175) of the mutant GnRHR protein (**Figure 2A**). Genetic approaches, albeit effective, are somehow impractical for *in vivo* application because the mutation could be directly corrected by modifying the corresponding DNA gene sequence (178).

Physical approaches to rescue misfolded proteins include incubating the cells at lower (*e.g.* 28-32 °C) than the physiological temperature. Lower incubation temperatures has resulted in enhanced PM expression of several conformationally defective vasopressin 2, GnRH, and FSH receptors with distinct point mutations (16, 176) (**Figure 2C**). It seems that for some misfolded receptors that are temperature-sensitive, the mechanism of this rescuing effect (75, 141, 179–182) is by preventing association and/or aggregation of the conformationally abnormal receptor with inhibitory chaperones (e.g. HSP90) (19, 183, 184), allowing the misfolded protein to be exposed to those mechanisms that favor processing and anterograde trafficking through the secretory pathway (185).

Chemical rescue is a general mechanism through which cell surface PM expression of misfolded proteins may be achieved (186) Chemical chaperones (e.g. non-specific stabilizing agents like polyols and sugars) are low molecular weight compounds that do not interact with the misfolded proteins or interefere with their function, and that correct folding by stabilizing their conformation. Osmolytes stabilize misfolded proteins by reducing their free movement and increasing their hydration (186), thereby preventing aggregation of immature conformers. They modify the free-energy difference between partially folded and more compact native structures. Since high concentrations of chemical chaperones are required to be effective in promoting folding of mutant proteins they are too toxic as an *in vivo* therapeutic strategy. Since chemical chaperones unspecifically rescue misfolded proteins, the risk exists of increasing retention in different cellular compartments or enhancing secretion of proteins unrelated to the mutant one, thereby leading to inappropriate changes in their local levels and/or secretion that may compromise cell function (187). As an exception, TMAO (trimethylamine-N-oxide), glycerol, and 4-phenylbutyric acid, may increase the secretion efficiency of misfolded α1-antitrypsin in a selective manner, without an impact on levels of other proteins or proteasomal degradation (188).

Rescue of intracellularly trapped GPCRs can also be achieved through the manipulation of the ER and/or post-ER regulatory mechanisms involved in protein export. For example, in the P23H mutation in the rhodopsin gene, which leads to autosomal dominant retinitis pigmentosa due to rhodopsin misfolding, overexpression of BiP/Grp78 may reduce photoreceptor apoptosis and retinal degeneration, thereby allowing recovery of retinal function in rats (189). Another strategy to manipulate the QCS to correct misrouting is by using cell-penetrating peptides, which modify cytosolic Ca^2+^ stores and, consequently, impact on Ca^2+^-regulated chaperones that regulate post-ER quality control (93). However, similar to chemical chaperones, a major problem with these approaches is their poor specificity for the target protein.

Molecular chaperoning has been a tremendously useful paradigm to develop pharmacoperones (or pharmacochaperones), which may regulate with high specificity folding and intracellular trafficking of WT and mutant, misfolded proteins (**Figure 1B**). In contrast with the poor specificity of chemical chaperones (190) (which is similar to that exhibited by a number of molecular chaperones), small molecule pharmacoperones have several advantages: *a.* they exhibit highly selective binding to the conformationally abnormal protein; and *b*. they do not interfere with the degradation of other misfolded proteins that are normally cleared by the QCS (191, 192). Pharmacoperones serve as a molecular template to help misfolded proteins to properly fold and become more stable, minimal free-energy conformers that may pass the scrutiny of the QCS (193). Of course, the efficiency of pharmacoperones to stabilize the target protein and restore its PM expression and function relays on several factors, including their specific structure and mode of action (either as agonist, antagonist of the natural ligand or allosteric modulator of the receptor, which in turn determine the selectivity towards the target receptor), the degree of the folding defect, and the specific location of the mutation which should not involve motifs essential for receptor function (see above) (194).

For instance, human V_2_R mutants (which cause X-linked nephrogenic diabetes insipidus) displaying amino acid exchanges at the TMD2 and TMD4 interface (H80R, W164R, and S167L mutants), are recalcitrant to pharmacoperones, probably because the replaced residues provoke a severe folding defect (195). This is also the case of the human GnRHR S168R and S217R mutants at the TMDs 4 and 5, which cause congenital hypogonadotropic hypogonadism; replacement of any of these serine residues (at the lipid membrane-contact phase of those TMDs) with arginine (which is a highly hydrophilic amino acid) provokes a thermodynamically unfavorable exchange that causes rotation of the TMDs 4 and 5, thereby changing the orientation of the EL2, making extremely difficult the formation of the C14-C200 disulfide bridge, which in this particular receptor is essential for receptor trafficking and function (17, 111). Mutational defects that interfere with agonist binding would also display limited *functional* recovery of the misfolded receptor in response to pharmacoperones, despite the successfully increased cell surface PM expression of the mutant receptor.

## Pharmacoperones and functional rescue of misfolded GPCRs

Pharmacological chaperones are molecules that correct folding of misfolded proteins preventing their aggregation and/or degradation, thereby facilitating escape of the abnormal protein from the QCS to correct misrouting. Characteristics of pharmacoperones for *in vivo* administration should include at least the following features (193):

- To easily reach physiologically effective concentrations with minimal side effects when administered *in vivo*.
- To easily cross the cell surface PM and bind with high specificity the misfolded protein, primarily in the ER, but also in other intracellular compartments (**Figure 1B**).
- To localize and act where the misfolded protein is intracellularly retained.
- To exhibit high specificity and enough residence time in the ER or other post-ER compartment so that mutants can be effectively rescued, and
- To bind the target protein in a reversible manner, facilitating its dissociation after routing the stabilized receptor to its functional location, to prevent any competition with the endogenous ligand, particularly in the case of agonist and antagonist pharmacoperones.

Antagonist pharmacoperones aid the misfolded GPCR mutant to reach its minimal energy, and stabilize its native conformation allowing trafficking of the protein to the PM. Nevertheless, pharmacoperone antagonists should be removed after promoting receptor trafficking and PM expression so that it may be recognized by and bind its cognate ligand (194). Interestingly, *in vivo* studies administering the antagonist IN3 to male mice harboring the E90K mutation did not require an additional maneuver to rescue function of the misfolded receptor other than administration of the pharmacoperone in a pulsatile manner, so that during the inter-pulse intervals the antagonist can be “washed” from the rescued receptor (196, 197). Ideally, antagonist pharmacoperones should be effective at the lowest concentration, so that it may easily dissociate from the rescued receptor (198).

Unlike antagonists, agonist pharmacoperones do not need to dissociate from the receptor protein after rescue, albeit the potential problem of promoting receptor desensitization and internalization should be considered since this may provoke decreased response to the endogenous ligand. Interestingly, some particular pharmacoperones with agonist acitivity may promote rescue and PM expression of misfolded human V_2_R mutants and enhance arginine vasopressin-stimulated cAMP signaling without promoting β-arrestin recruitment, ligand/receptor internalization, and β-arrestin-mediated mitogen activated protein kinase (MAPK) activation, similar to the effects of biased agonists (199), which may have some therapeutic implications because of their selective effect on a particular function. Finally, allosteric ligand pharmacoperones (see below) are compounds that specifically bind the receptor protein at a site different from the orthosteric binding site of the receptor, and thus do not interfere with binding of the natural ligand, albeit positive allosteric modulators may also lead to increased desensitization/internalization of the rescued receptor.

The efficacy of pharmacoperones to prevent abnormal intracellular accumulation and rescue misfolded GPCRs has been demonstrated for a number of receptors, including those associated with endocrine disease (**Table 1**). Some of these misfolded GPCR mutants leading to endocrine disorders have shown functional rescue in response to different pharmacoperone molecules *in vitro* and *in vivo*. Some examples follow.

- In patients with X-linked nephrogenic diabetes insipidus (NDI), concentration of urine by the kidney is not possible due to defects in the V_2_R, which is localized in the basolateral membrane of principal cells in the renal collecting duct (142, 200). Occupancy of the V_2_R by its ligand, arginine-vasopressin (AVP), stimulates translocation and exocytic insertion to the luminal membrane of the water channel protein aquaporin-2, leading to water reabsorption in the kidney (201). Mutations in the V_2_R are associated with the development of NDI, a disease in infants that has an unfavorable prognosis if untreated early in life (142, 202–204). Given that a number of mutant V_2_Rs can be included in Class II GPCR mutants (18, 75), they can be rescued either by genetic or pharmacologic means. In this regard, it has been shown that a number of PM permeable antagonists may specifically bind and rescue function of several misfolded, trafficking-defective V_2_R mutants in *in vitro* conditions (110). V_2_R non-peptide antagonists include SR49059 (relcovaptan, which is a V_1A_ receptor antagonist exhibiting moderate affinity for the V_2_R), OPC31260, OPC41061 (tolvaptan), and SR121463B (satavaptan). These pharmacoperones have been shown to promote maturation and basolateral membrane localization of some V_2_R mutants stably transfected in MDCK cells (143). In a proof-of-concept *in vivo* study, the effects of the V_1A_R/V_2_R antagonist SR49059 to rescue function of three V_2_R mutants (R137H, W164S, and des185-193 V_2_R mutants) expressed by young patients with NDI were studied (110). Urine production and water intake decreased, and concomitantly urine osmolarity was significantly enhanced in response to this compound. More clinically safe than SR49059 (which is hepatotoxic) for rescuing misfolded V_2_Rs at feasible concentrations *in vivo* are the pharmacoperones OPC31260 and OPC41061, which represent promising candidates to specifically treat NDI (143).
- Contrary to pharmacoperone antagonists, pharmacoperone agonists activate the cAMP/PKA signaling cascade after binding to V_2_R mutants. It has been shown that the non-peptide agonists MCF14, MCF18, and MCF57 may selectively rescue cell surface plasma membrane expression and restore the response to AVP of several V_2_R mutants, including L44P, A294P, and R337X misfolded V_2_Rs, without stimulating receptor internalization and MAPK activation (199, 205). On the contrary, the cell membrane-permeable agonists VA999088, VA999089, and OPC51803 only stimulated intracellular signaling but not PM expression (144). Analogously to the S168R and S217R human GnRHR mutants, the V_2_Rs with amino acid exchanges at the TMD2 and TMD4 interface (H80R, W164R, and S167L mutants) also showed recalcitrance to pharmacoperone treatment, most probably due to the severity of the folding defect as a consequence of the nature of the amino acid replacement (111, 195). These data underline the importance of the location of the amino acid substitution, the physicochemical properties of the replacing residue, and the nature of the pharmacoperone drug administered on the response of misfolded V_2_R mutants (and also of other GPCRs). Finally, it has been shown that the aminoglycoside antibiotic (G418) can rescue X-linked NDI caused by a V_2_R mutant bearing a premature truncation (206). Cultured kidney collecting duct cells expressing the V_2_R E242X mutant and exposed to this particular antibiotic exhibited enhanced AVP-stimulated cAMP responses. In addition, G418 suppressed the premature stop codon present in mice bearing the E242X replacement; in this model, mRNA translation proceeded effectively to the normal end of the gene, improving urine concentration. In addition, it has also been shown that stop codons can be suppressed by aminoglycosides *in vitro* and *in vivo* (207).
- MC3R and MC4R are GPCRs mainly expressed in the central nervous system and involved in energy homeostasis, regulating food intake and energy expenditure (75, 163, 208); mutations in their corresponding genes lead to early-onset severe obesity. A large number of mutant MC3 and MC4 receptors correspond to Class II loss-of-function mutants. Several MC4R Class II loss-of-function mutants may be rescued by pharmacological chaperones *in vitro* and *in vivo*. For example, the small molecule ML00253764, which is a MC4R antagonist and partial inverse agonist permeable to the blood-brain barrier, enhanced cell surface PM expression and ligand-stimulated cAMP response of mutant MC4Rs *in vitro* (163, 209). Nonetheless, this molecule has a relatively high EC_50_ rescue dose (∼10 μM), which is not practical for *in vivo* rescue. In contrast, Ipsen 5i and Ipsen 17 are more potent pharmacoperones for MC4R mutants (EC_50_ dose approximates 10 nM) (210); in fact, the former has been shown to correct misrouting and signaling of intracellularly trapped MC4R mutants (75, 162). More recently, *in vivo* studies in humanized mouse models bearing the R165W human MC4R mutant showed that UM013086 rescued PM expression and function of this obesity-causing mutant MC4R in mice; treated animals restored the anorexigenic response to the MC4R agonist melanotan II (211). This study represents a proof-of-principle for pharmacoperone application as a therapeutic strategy for severe obesity associated with mutations in the MC4R.
- The CaSR is a GPCR essential to maintain blood Ca^2+^ homeostasis by sensing fluctuations of extracellular Ca^2+^, modulating parathyroid hormone secretion by parathyroid cells and Ca^2+^ reabsorption by the kidney (153, 154). Loss-of-function of the CaSR caused by heterozygous mutations in this receptor gene leads to familial hypocalciuric hypercalcemia, whereas homozygous mutations cause severe neonatal hyperparathyroidism (155). A number of CaSR loss-of-function mutations (*e.g.* R66C, R185Q, R680C, R795W, and V817I, which affect proper trafficking of the CaSR through the secretory pathway), can be partially or completely rescued *in vitro* by the membrane-permeant allosteric agonist NPS R-568 or MG132 (which prevents proteasomal degradation) (156, 212). The finding that the location and function of inactivating CaSR misfolded mutants can be rescued by pharmacoperones, opens the possibility for novel therapeutic approaches for diseases caused by structural alterations leading to misrouting of this important receptor.
- Hypogonadotropic hypogonadism (HH) in humans may originate at different levels along the hypothalamic pituitary unit (73, 213). Kallmann syndrome is an X-linked recessive inherited disease whose phenotype includes delayed puberty and anosmia or hyposmia due to failure of GnRH producing neurons to migrate from the olfactory placode to the basal hypothalamus and to the presence of insufficient projections from the lateral olfactory placode to the forebrain, resulting in aplasia or hypoplasia of the olfactory bulbs. Mutations in the PROKR2, some of which provoke misfolding and misrouting of the receptor, may be associated with this syndrome (150, 214). In this particular receptor, the small molecule PROKR2 antagonist A457 has been shown to rescue PM expression and signaling of the misrouted P290S PROKR2 mutant (150).
- Other mutations causing hypogonadotropic hypogonadism, without affecting olfaction, include mutations in the GnRHR (73, 149, 215, 216). This receptor binds GnRH, a hypothalamic decapeptide released into the median eminence at the base of the hypothalamus, which serves as an interface between the neural and the endocrine system governed by the anterior pituitary (217). Here, several hypothalamic hormones, including GnRH, are released into the portal capillary bed to be transported to the anterior pituitary. GnRH binds to the GnRHR to promote the synthesis and secretion of the pituitary gonadotropins, which are essential for gonadal function. The molecular mechanisms through which small molecules rescue misfolded human GnRHRs has been dilucidated with some detail (46, 191, 198). Using a number of methods including site-directed mutagenesis, confocal microscopy, computational modeling, and pharmacoperone docking, it has been possible to elucidate the mechanism whereby distinct pharmacoperones (*e.g*. the indole IN3) stabilize misfolded GnRHRs (191). In the E90K mutant (and probably in other GnRHR mutants as well), whose PM expression and functional rescue *in vitro* is complete when pharmacological approaches are applied (see above) (173), this effect occurs through the association of residues D98 and K121 (at the extracellular face of the TMD1 and at the TMD3, respectively) *via* formation a surrogate bond for the highly conserved, naturally occurring E90-K121 salt bridge; the pharmacoperone IN3 leads to stabilization of the TMD2-TMD3 configuration which has been severely altered by the E90K substitution (191). Notably, all GnRHR pharmacoperones tested, including indoles, the quinolone Q89, and two erythromycin macrolides, A177775 and TAK-013, which were originally developed as GnRH peptidomimetic antagonist molecules, rescued function of the majority of the human GnRHR mutants studied *in vitro*, including the T32I, E90K, C200Y, C279Y, and L266R GnRHR mutants identified in patients with HH (198, 218), despite their different distribution accross the receptor protein. This finding indicates that the orientation between the TMD2 and 3 is a critical structural feature monitored by the QCS of the cell (17, 191, 198). Even more importantly, employing a HH murine model expressing the E90K GnRHR mutation (178), it was demonstrated that pulsatile administration of IN3 was able to induce changes in several biomarkers, of hypogonadism, including restoration of spermatogenesis, providing evidence on the efficacy of pharmacoperone treatment on this mutant receptor (196).
- Loss-of-function mutations in the LHCGR and the FSHR may lead to hypergonadotropic hypogonadism in humans (73, 219, 220), whenever both alleles are affected by the mutation. Loss-of-function mutations in the LHCGR gene lead to different phenotypes in male patients, from severe genital ambiguity to cryptorchidism and micropenis, whereas women with inactivating mutations in this receptor frequently exhibit primary or secondary amenorrhea and infertility (73). In men, loss-of-function mutations in the FSHR gene, may cause decrease in the quality of spermatogenesis but with normal testosterone production; this latter feature probably contributes to the preservation of fertility exhibited by some patients bearing these mutations (221). In women, inactivating mutations in the FSHR lead to more severe phenotypes, mainly varying forms of premature ovarian failure (222–224). Approximately fifty percent of loss-of-function mutations in the LHCGR and ∼30% in the FSHR are misfolded, trafficking defective proteins which fail to be recruited to the secretory pathway (185). Exposure of cells expressing the A593P and S616Y misfolded LHCGR mutants (which cause in men varying degrees of genital ambiguity due to hypoplasia of testicular interticial cells; **Table 1**) to the cell-permeant, small molecule agonist Org 42599 rescued PM expression and function of these LHCGR misfolded mutants (225),whereas in misfolded FSHR mutants, *in vitro* exposure to the thienopyr(im)idine Org41841 (226) or to the FSHR allosteric agonist CAN1404 (a dihydrobenzoindazole analogue) resulted in increased PM expression and function of some misfolded mutants [*e.g.* the A189V mutant (at the receptor ectodomain) by Org41841, and the D408Y, A419T, A575V, P587H, P519T, and F591S FSHR mutants (at the serpentine region of the receptor) by CAN1404] (170, 171).

## Conclusions

Current challenges in the area of targeting trafficking of misfolded proteins as a pharmacological approach include identification of novel small molecules that may effectively exert its therapeutic effect *in vivo* to cure conformational diseases, including those due to GPCR misfolding. It is also important to discover molecules that may increase PM expression of WT receptors and provoke conformational bias with positive effects on GPCR-signalosome assembly and signaling upon exposure to agonist (227). Assays to identify pharmacological chaperones lacking antagonistic activity are currently available (172, 228–234), but nevertheless, new assays are still needed to bypass the complex pharmacological interactions occurring when both antagonist and conformational biased activities are present.

An example of pharmacoperones as a feasible therapeutic approach to ameliorate the effect of misrouting is the case of the misfolded ΔF508 CFTR chloride channel leading to cystic fibrosis (235, 236). This CFTR mutant is retained by the chaperone HSP70 and its co-chaperone CHIP in the ER and retro-translocated into the cytosol for degradation by the ubiquiting-proteasome machinery (237–239). Nevertheless, in the presence of small molecule correctors, the ΔF508 CFTR mutant may route to the PM and exert function. An additional problem is that the peripheral QCS of the cell may recognize this particular mutant when is present at the PM and accelerate its internalization, thereby enhancing its lysosomal degradation, which may reduce the net amount of functional CFTR molecules at the PM. To overcome this problem, combination of modulators (correctors and potentiators) of this channel, which may be considered as pharmacoperone drugs, have been succesfully administered to patients with cystic fibrosis bearing the ΔF508 CFTR mutation (25, 27, 30, 240–246), resulting in a significant improvement on some primary and secondary end points (30, 242). Small molecule modulators of CFTR are thus highly promising therapeutic interventions for patients with cystic fibrosis, particularly those expressing the ΔF508 mutation.

It is currently possible to obtain valuable information on the mechanism of action of different chemical classes of small molecule pharmacoperone drugs and how they interact with the misfolded GPCRs, applying high-throughput screening assays coupled with virtual computed, artificial intelligence-aided structural approaches (172, 247–253). This might provide new medicines to cure endocrine diseases caused by mutations leading to misfolding and misrouting of GPCRs.

## Author contributions

AU-A: Write the manuscript. TZ: Write the manuscript and provided data. RG-S: Write the manuscript. Y-XT: checked the manuscript. All authors contributed to the article and approved the submitted version.

## Funding

Research in the authors’ laboratories are supported by grants from the National University of Mexico (UNAM-CIC) (to AU-A).

## Conflict of interest

The authors declare that the research was conducted in the absence of any commercial or financial relationships that could be construed as a potential conflict of interest.

## Publisher’s note

All claims expressed in this article are solely those of the authors and do not necessarily represent those of their affiliated organizations, or those of the publisher, the editors and the reviewers. Any product that may be evaluated in this article, or claim that may be made by its manufacturer, is not guaranteed or endorsed by the publisher.
